# Does culture affect How Mental Health Is Treated And Diagnosed- What Is The Future Of It

**DOI:** 10.1192/j.eurpsy.2022.370

**Published:** 2022-09-01

**Authors:** C. Musolo, J. Beezhold

**Affiliations:** 1 Sir Isaac Newton Sixthform, N/a, EL, United Kingdom; 2 Norfolk and Norwich University Hospital, Mental Health Liaison Service, Norwich, United Kingdom; 3 Hellesdon Hospital, Yare Ward, Norwich, United Kingdom; 4 University of East Anglia, Norwich Medical School, Norwich, United Kingdom

**Keywords:** psychiatry, Cultural, cross cultural

## Abstract

**Introduction:**

Culture is used to refer to the aspects of thinking, feeling, and behaviour related to nation, heritage, place of birth and ethnicity. I look at how the cultural context of mental disorders and the cultural context of mental disorders and the challenges of addressing ethnic diversity in psychiatric services because there is an over-representation of black people detained under the MHA.

**Objectives:**

My aim is to understand what current data shows and use this to find a way forward which identify issues with culture and independently and challenge policies, services systems and address culture in clinical practice to provide culture complement care.

**Methods:**

**Figure:**
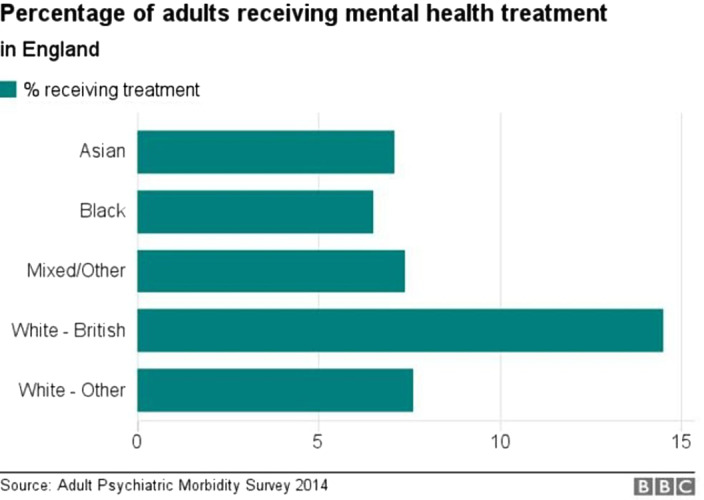

**Figure:**
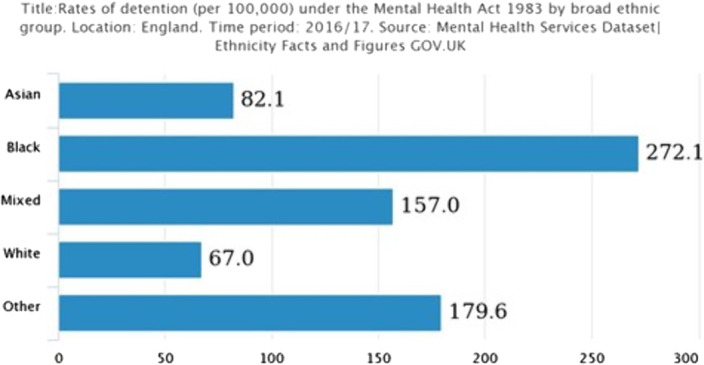

. research on rates of detention

**Results:**

The MHA acts tells us POC are 4 times more likely to be detained, arrested under 136 twice as much, and are 8 times as likely to be put on CTOS. 40% of black people will more likely asses care through the police system. (mind.org, Uk) This further shows us just how unrepresented POCs are when it comes to their diagnosis, treatment, and care, especially compared to their white counterparts.

**Conclusions:**

In conclusion regulatory bodies and clinicians have to work towards understanding and identifying the reasons for these disparities and then implementing measures to address this. Such as putting people of color in higher positions in mental health positions in mental health positions to add diversities, also teaching the staff members and other people in high positions of power how much culture really impacts mental health, culturally appropriate advocacy, and improving research done.

**Disclosure:**

No significant relationships.

